# Vaccine effectiveness against referral to hospital after SARS-CoV-2 infection in St. Petersburg, Russia, during the Delta variant surge: a test-negative case-control study

**DOI:** 10.1186/s12916-022-02509-8

**Published:** 2022-09-20

**Authors:** Anton Barchuk, Mikhail Cherkashin, Anna Bulina, Natalia Berezina, Tatyana Rakova, Darya Kuplevatskaya, Oksana Stanevich, Dmitriy Skougarevskiy, Artemiy Okhotin

**Affiliations:** 1 Institute for Interdisciplinary Health Research, European University at St. Petersburg, Shpalernaya Ulitsa 1, St. Petersburg, 191187 Russia; 2 Medical Institute named after Berezin Sergey, Esenina Ulitsa 2-3a, St. Petersburg, 194354 Russia; 3 Tarusa Hospital, Karla Libknekhta Ulitsa 16, Tarusa, 249100 Russia; 4 ITMO University, Kronverksky Prospekt 49, St. Petersburg, 197101 Russia

**Keywords:** Vaccine effectiveness, Case-control study, SARS-CoV-2, COVID-19, Pneumonia

## Abstract

**Background:**

The results of a randomised trial showed the safety and efficacy of Gam-COVID-Vac against COVID-19. However, compared to other vaccines used across the globe, the real-world data on the effectiveness of Gam-COVID-Vac, especially against the disease caused by the Delta variant of concern, was limited. We aimed to assess the effectiveness of vaccination mainly conducted with Gam-COVID-Vac in St. Petersburg, Russia.

**Methods:**

We designed a case-control study to assess the vaccine effectiveness (VE) against referral to hospital. Self-reported vaccination status was collected for individuals with confirmed SARS-CoV-2 infection who were referred for initial low-dose computed tomography (LDCT) triage in two outpatient centres in July 3–August 9, 2021, in St. Petersburg, Russia. We used logistic regression models to estimate the adjusted (for age, sex, and triage centre) VE for complete (14 days or more after the second dose) vaccination. We estimated the VE against referral for hospital admission, COVID-19-related lung injury assessed with LDCT, and decline in oxygen saturation.

**Results:**

In the final analysis, 13,893 patients were included, 1291 (9.3%) patients met our criteria for complete vaccination status, and 495 (3.6%) were referred to hospital. In the primary analysis, the adjusted VE against referral to hospital was 81% (95% confidence interval: 68–88) for complete vaccination. The VE against referral to hospital was more pronounced in women (84%, 95% CI: 66–92) compared to men (76%, 95% CI: 51–88). Vaccine protective effect increased with increasing lung injury categories, from 54% (95% CI: 48–60) against any sign of lung injury to 76% (95% CI: 59–86) against more than 50% lung involvement. A sharp increase was observed in the probability of hospital admission with age for non-vaccinated patients in relation to an almost flat relationship for the completely vaccinated group.

**Conclusions:**

COVID-19 vaccination was effective against referral to hospital in patients with symptomatic SARS-CoV-2 infection in St. Petersburg, Russia. This protection is probably mediated through VE against lung injury associated with COVID-19.

**Supplementary Information:**

The online version contains supplementary material available at (10.1186/s12916-022-02509-8).

## Background

Vaccination proved to be an effective pharmaceutical control measure during the COVID-19 pandemic. More than 4 billion doses have been already administered, but real-world evidence is not equally available for all vaccines used globally. Even though the results from clinical trials of Gam-COVID-Vac [1, 2] suggested safety and efficacy similar to other COVID-19 vaccines used in population-based programmes across the globe [3, 4], this finding was met with scepticism and criticism [5–9]. Despite adoption of Gam-COVID-Vac in 49 countries, the independent safety data for this vaccine is available only from San Marino and Argentina [10, 11]. The real-world evidence on Gam-COVID-Vac vaccine effectiveness (VE) from population-based studies is also limited [12–16].

A case-control study design and its modifications are the core methods to assess the effectiveness of vaccination in real-world settings [17]. Multiple case-control studies of COVID-19 vaccines have been published [18, 19]. The study that explored the VE of vaccines available in Scotland showed diminished protection against the Delta variant of concern (VOC) infection and hospital admission [20]. The COVID-19 pandemic in Russia has claimed more than 500,000 excess deaths by the spring of 2021 [21], while the lack of real-world evidence on the VE became a public health issue in Russia. Vaccination uptake in the country was undermined by vaccine hesitancy [22], partly driven by the lack of independent research exploring vaccine safety and effectiveness and failures to communicate the balance of benefits and harms of COVID-19 vaccination.

St. Petersburg is the second most populated city in Russia and the fourth in Europe. More than 45% of the population have contracted the SARS-CoV-2 infection by the end of April, 2021 [23]. However, only 10% received at least one dose of any vaccine by April, 2021, which is below the levels seen in other countries [23]. Low uptake by the spring of 2021 failed to stop the spread of the new Delta VOC in May–June 2021 in St. Petersburg. The spread of the Delta VOC, in turn, caused the rise in vaccination demand in the summer of 2021. As reported by the city government, by July 23, 2021, approximately 27% of the adult population of St. Petersburg received at least one dose of any vaccine, and 19% received two doses [24]. Consequently, this increase in the vaccination uptake has led to an increase in the absolute number of vaccinated individuals who reported to have a breakthrough infection or hospital admission. Such anecdotal evidence has caused further mistrust in the vaccination programme without reliable scientific reports.

This paper presents the first independent assessment of the VE in Russia. We designed a case-control study to assess the VE against referral to hospital in the individuals who reported symptoms and were referred for initial computed tomography assessment in two outpatient centres in St. Petersburg, Russia.

## Methods

### Population and study design

St. Petersburg and other cities in Russia have established a triage service for symptomatic patients with COVID-19. Symptomatic patients with confirmed SARS-CoV-2 (using polymerase chain reaction (PCR) test) are referred to outpatient triage, including brief physical examination and low-dose computed tomography (LDCT). The decision about hospital admission is based on symptoms, i.e. shortness of breath, oxygen saturation, overall clinical condition, and lung injury assessed with LDCT. We aimed to determine the VE against hospital referral and COVID-19 lung injury in symptomatic patients with confirmed SARS-CoV-2 infection, who were referred to the LDCT triage. VE in our study is defined as the reduction in odds of several severe outcomes (hospitalisation and lung injury) in patients with confirmed COVID-19, and it does not approximate the VE against infection or severe outcomes in comparison with healthy controls. We used a test-negative case-control study design, which is also defined as case-control with “other patient” control [25]. This study design is traditionally used to estimate VE against infection. However, in several instances, it was also used to estimate the protection or harmful effect of drugs against severe disease in symptomatic patients [26, 27].

We retrospectively collected individual-level patient data from two outpatient triage centres of the Medical Institute named after Berezin Sergey (MIBS), a private medical facility contracted by the city government to provide triage service for nearly half of the city districts. All patients referred to LDCT triage underwent brief physical examination, including pulse oximetry. A local computed tomography score (CT-score) was implemented in Russia [28]. It has five gradations (0, 1, 2, 3, 4) which are related to the volume of involved lung segments (0, less than 25%, 25–50%, 50–75%, 75–100%). It is used in the country to define the severity of COVID-19 lung injury and to triage patients. CT-score of 3 and 4 mean more than 50% of lung volume involvement and is often used as an indication for hospital admission. Patients with CT-score less than 3, i.e. less than 50% lung injury seen on the LDCT, and in the absence of severe symptoms, are normally sent to out-patient treatment or follow-up.

### Vaccination status and outcomes

The information about self-reported vaccination status was collected at the time of appointment to the LDCT. The patients reported the number of doses and the dates of vaccination, the type of vaccine data was not collected. The majority of vaccinated residents in St. Petersburg received Gam-COVID-Vac. Other vaccines used in Russia were EpiVacCorona and CoviVac, but efficacy studies for them are not available. The proportion of the population that received other vaccines was 10% or less in St. Petersburg [16, 24].

We used several definitions of the vaccination status. Complete vaccination status was assigned to the patients who had reported receiving two doses at least 14 days before the referral to the LDCT triage. Partial vaccination status was assigned to the patients who failed to meet the above criteria for complete vaccination, but had reported receiving one dose at least 14 days prior to the referral. In addition, for the complete vaccination status, we stratified the period after the second dose to the period of 14 to 55 days and the period of 56 or more days.

In our primary analysis, cases were all patients referred to hospital after triage starting from July 3, 2021, till August 9, 2021, during the Delta VOC surge in St. Petersburg. Controls were the patients referred to outpatient treatment and follow-up after the LDCT triage in the same period.

In the secondary analyses, we used the CT-score [28] and oxygen saturation as the secondary outcomes to assess the VE against COVID-19 lung injury. Oxygen saturation was grouped in the ranges according to the NEWS2 score [29], which was well-validated in non-COVID-19 settings. It has four gradations (0, 1, 2, 3) which are related to the oxygen saturation in the following categories: more than 95, 94–95, 92–93, less than 92.

### Statistical analysis

We modelled our study plan following the WHO interim guidance to evaluate COVID-19 vaccine effectiveness [30]. We collected information on all patients referred to the LDCT triage, as most patients were sent for out-patient treatment and outpatient follow-up. We used unconditional logistic regression for our primary and secondary outcomes to estimate odds ratios (ORs) for vaccination status among cases and controls, which approximates ORs for the outcomes (hospital admission, different levels of lung injury, and decline in oxygen saturation) among the vaccinated and non-vaccinated patients. For several levels of the outcome (e.g. lung injury categories), we used several models with binary outcomes sharing the same reference group (e.g. patients without any lung injury). The VE was calculated as 100*%*×(1−*OR*) adjusted for age (continuous variable), sex, and the triage LDCT centre. We abstained from post hoc sample size calculations [31].

All standard errors and confidence intervals were adjusted for heteroskedasticity with the Huber-Eicker-White sandwich estimator. Finally, we investigated the relationship between the outcomes and the thin plate regression spline of the patient age by vaccination status in a semiparametric logistic regression.

## Results

Overall, 13,893 patients were included in the final analysis. Patient characteristics are presented in Table 1. Among all patients, 1964 (14.1%) received at least one dose, 1379 (9.9%) received two doses, 1291 (9.3%) met our criteria for complete vaccination status, and additionally, 448 (3.2%) met our criteria for partial vaccination status (one dose at least 14 days prior to the referral). Four hundred ninety-five (3.6%) patients were referred to hospital after the LDCT triage (232 or 4.1% from the one outpatient triage centre and 263 or 3.2% from the other). The majority of referred patients (63.1%) had CT-score 3–4 or > 50% lung involvement on LDCT. Patients referred to hospital were also older (66.1% were older than 60 years). Only 17 (3.4%) patients who were referred for hospital admission met the criteria for complete vaccination.

**Table 1:** Characteristics of patients with COVID-19 referred to triage centres

		Overall	Referred to outpatient	Referred to
			Follow-up (%)	Hospital (%)
		13,893	13,398	495
Age (mean (SD))		48.2 (15.9)	47.6 (15.6)	64.9 (14.6)
Age (categories (%))	18–30	2050 (14.8)	2042 (15.2)	8 (1.6)
	31–40	2961 (21.3)	2931 (21.9)	30 (6.1)
	41–50	2746 (19.8)	2698 (20.1)	48 (9.7)
	51–60	2,750 (19.8)	2668 (19.9)	82 (16.6)
	60+	3386 (24.4)	3059 (22.8)	327 (66.1)
Sex (%)	Female	8585 (61.8)	8,270 (61.7)	315 (63.6)
	Male	5308 (38.2)	5128 (38.3)	180 (36.4)
Triage centre (%)	1	5655 (40.7)	5423 (40.5)	232 (46.9)
	2	8238 (59.3)	7975 (59.5)	263 (53.1)
Vaccine doses (%)	None	11,929 (85.9)	11,464 (85.6)	465 (93.9)
	1 dose	586 (4.2)	573 (4.3)	13 (2.6)
	2 doses	1,378 (9.9)	1361 (10.2)	17 (3.4)
Vaccination status (%)	Non-vaccinated	12,154 (87.5)	11,687 (87.2)	467 (94.3)
	Partial	448 (3.2)	437 (3.3)	11 (2.2)
	Complete	1291 (9.3)	1274 (9.5)	17 (3.4)
Oxygen saturation (%)	96% and more	13,508 (97.2)	13,348 (99.6)	160 (32.3)
	94–95%	152 (1.1)	9 (0.1)	143 (28.9)
	92–93%	140 (1.0)	1 (0.0)	139 (28.1)
	Less than 92%	53 (0.4)	2 (0.0)	51 (10.3)
	Missing data	40 (0.3)	38 (0.3)	2 (0.4)
Lung injury (%)	No injury	4525 (32.6)	4525 (33.8)	0 (0.0)
	Less than 25%	7638 (55.0)	7638 (57.0)	0 (0.0)
	25–50%	1415 (10.2)	1232 (9.2)	183 (37.0)
	50–75%	284 (2.0)	3 (0.0)	281 (56.8)
	More than 75%	31 (0.2)	0 (0.0)	31 (6.3)

In the primary analysis, the adjusted VE against referral to hospital admission was 81% (95% confidence interval: 68–88) for complete vaccination (Table 2). The effect of the partial vaccination against referral to hospital was 35% (95% CI: − 21–65).

**Table 2:** Effectiveness of complete vaccination against referral to hospital, overall and according to age group, sex, and triage centre

		Referred to	Referred to	Crude VE	Adjusted VE
		Outpatient follow-up*	Hospital*	(95% CI)	(95% CI)
Overall		1,274/12,124	17/478	66% (45–79)	81% (68–88)
Sex	Female	752/7,518	8/307	74% (47–87)	84% (66–92)
	Male	522/4,606	9/171	54% (9–76)	76% (51–88)
Age	18–49	426/6,997	2/77	57% (-74–90)	63% (-51–91)
	50 and older	848/5,127	15/401	77% (62–87)	82% (69–89)
Triage centre	1	456/4,967	7/225	66% (28–84)	79% (54–90)
	2	818/7,157	10/253	65% (35–82)	82% (64–91)

^∗^Completely vaccinated/non-vaccinated and partially vaccinated

The crude vaccine effect again any lung injury was 36% (28–43) and 54% (48–60) after adjustment for age, sex and triage centre. VE estimates against other outcomes related to severe diseases were in line with the main results. Crude and adjusted VE against severe lung injury were 58% (31–74) and 76% (59–86). Crude and adjusted vaccine effect against less than 96% oxygen saturation were 53% (22–72) and 70% (49–82). Only a few patients had more than 75% of lung involvement and none in the vaccination group, so we calculated the VE for the combined category, which included patients with more than 50% of lung involvement.

**Fig. 1:**
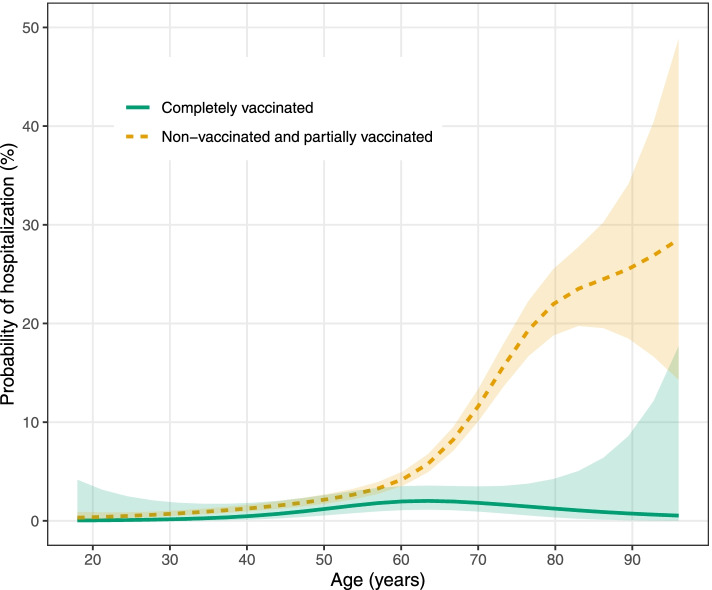
Probability of referral to hospital, according to age and vaccination status (shaded areas are 95% CI)

The VE against hospital admission was more pronounced in women (84%, 95% CI: 66–92) compared to men (76%, 95% CI: 51–88) and older age groups (77%, 95% CI: 62–86) (Table 2). We observed a sharp increase in the probability of hospital admission with age for non-vaccinated patients in relation to an almost flat relationship between age and the probability of hospital admission for the completely vaccinated group of patients (Fig. 1 and Fig. A1 in Supplementary material). We observed an increase in the probability of any lung injury with age in both groups with similar VE estimates across different age groups (Figs. A2 and A3 in Supplementary material). The adjusted VE by the time elapsed from the second dose was 60% (95% CI: 5–83) for the 14–55 day period and 85% (95% CI: 72–92) for the 56+ days. There was no difference in the VE by the LDCT triage centre. Crude VE estimates were lower compared to the adjusted VE.

## Discussion

This is the first study examining the real-world effectiveness of COVID-19 vaccination in Russia and one of the first studies globally to provide information about the vaccine protective effect against COVID-19-associated lung injury. Vaccination showed 81% (95% CI: 68–88) protection against referral to hospital in patients with symptomatic SARS-CoV-2 infection during the third wave of COVID-19 pandemic caused by Delta VOC in St. Petersburg. It is similar to the VE against Delta VOC reported for other COVID-19 vaccines [20].

The clear strength of this study is the availability of independent lung injury assessment using the LDCT. The radiologists did not have information on the vaccination status, while the computed tomography was applied as a triage method for all symptomatic patients. The protective effect of vaccination was consistent against all grades of lung injury. We did not observe 75% or more lung tissue involvement during the LDCT in the group of fully vaccinated patients, suggesting a strong and consistent protective effect. The protective effect was also observed against the decline in oxygen saturation. It was clear that both secondary outcomes (lung injury and decline in oxygen saturation) are closely related, but the oxygen saturation and LDCT were independently evaluated at the triage centres. Both factors independently influence the decision to refer to hospital, thus explaining the greater VE against hospital admission than for CT-score and oxygen saturation alone. These results further support the findings of the primary analysis.

Since all the patients in our study had COVID-19, our results provide specific information on vaccination impact on disease severity and its clinical course. When breakthrough infections are evolving as a cause of major concern, our data is assuring that the vaccine effect is going beyond the risk of contracting the infection. It shows that vaccination significantly diminishes disease severity and protects the lungs from virus-induced injury.

While SARS-CoV-2 is likely converting from a pandemic virus to an endemic one, the role of vaccination is also evolving from pandemic control to damage control. Our data shows that it is feasible. The infection fatality rate for COVID-19 is progressively increasing with age in St. Petersburg [23]. Our data shows that it is an effective way of protecting the most vulnerable elderly persons from the devastating consequences of the pandemic.

Lung injury is not only observed in COVID-19 but also in many other respiratory viral infections [32]. Lung injury leading to pneumonia and acute respiratory distress syndrome is a major cause of morbidity and mortality in these diseases. Lung imaging with computed tomography was used for assessment and risk stratification in COVID-19 [33]. However, in the USA and Europe, computed tomography was discouraged by the professional societies as the primary imaging modality in mild COVID-19 due to high cost and transmission risk, with emphasis placed on clinical assessment instead [34]. In Russia, due to less developed primary care and the low cost of the LDCT, it became the primary imaging modality used for COVID-19 triage. This offered the unique opportunity to assess the VE against lung injury detected with LDCT.

Our study does not provide direct evidence on the VE against SARS-CoV-2 infection. However, some indirect inferences can be made when analysing the characteristics of the overall patient group in our study. By July 23, 2021, the city government reported that at least one dose of vaccine was given to 1,178,266 residents of St. Petersburg (26.5% of the adult population), and 819,768 received two doses (18.5% of the adult population) [24]. In our study, among 13,893 symptomatic patients referred to the LDCT triage, only 12.5% received at least one dose, and 9.3% received two doses. In the absence of individual-level data, we can only estimate crude effectiveness against the symptomatic disease at around 50%. Our following case-control study confirmed that VE against symptomatic disease is likely higher than the estimated 56% due to bias arising from the high prevalence of the past COVID-19 in St. Petersburg [16].

Partial vaccination yields especially unreliable VE estimates due to low prevalence. The VE against Delta VOC for one dose of vaccine was lower than for two doses, and the effect did not clearly manifest until at least 28 days after the first one [20]. We obtained similar results in our study. The point estimate for partial vaccination was 34%, but the confidence interval for the corresponding OR included unity, suggesting an inadequate sample size. In Russia, the recommended 21-day interval between the first and the second dose of Gam-COVID-Vac is usually strictly followed.

In contrast to another study [18], we did not find any indications of waning efficacy. The VE was higher in patients with a longer period after the second dose. However, these results should be interpreted with caution, as possible biases can intervene in the stratified analysis of observational data. It is also important to distinguish between protection against infection and severe disease.

There are several important limitations of our study. The small number of vaccinated prevents us from conducting an adequately powered stratified analysis or estimating VE for one dose. It is also making the estimates less precise than ideal. We did not have the information on the vaccine type, so the estimated VE represent an average effect of vaccination in St. Petersburg. However, in its response to an inquiry by a member of St. Petersburg Legislative Assembly, the city health committee revealed that, by July 23, 2021, in St. Petersburg, among 1,227,496 individuals who received at least one vaccine dose, 1,178,266 or 96% received Gam-COVID-Vac, and the rest is distributed between the other two vaccines (EpiVacCorona—21,943 or 1.7% and CoviVac 27,287 or 2.2%) [24]. The October 2021 survey showed that only about 10% of responders in St. Petersburg received a vaccine other than Gam-COVID-Vac [16]. It is safe to assume that our study approximates the effectiveness of Gam-COVID-Vac. The effectiveness for EpiVacCorona and CoviVac would be difficult to derive from observational case-control studies due to low uptake.

Another important limitation of our study is possible referral bias. Patients were referred to the triage centres if they had positive PCR tests and disease symptoms, but the decision to refer was left at the physician’s and patients’ discretion. We cannot rule out that the vaccination status influenced this decision. If this is the case, we would observe more severely ill vaccinated individuals than the unvaccinated ones in our data.

Alternatively, the physicians may fully rely on vaccine protection and never refer the vaccinated patients to the triage. However, we observed many vaccinated individuals at the triage centres. Therefore, we believe that the referral bias would result in the underestimation of the VE. Vaccination could also influence patients’ medical care-seeking behaviour. We assume that self-assurance on vaccine protection would predominantly prevent patients with a mild disease from seeking medical care. This would also lead to underestimating the VE against severe disease in our study. The study results may also be biased if the triage centre referred symptomatic patients to the hospital based on their vaccination status. To the best of our knowledge, this decision was solely based on physical examination and the LDCT results. The vaccination status was collected for research purposes and was not a part of any official medical records. However, we cannot rule out this bias completely. There was a slight difference between triage centres in the proportion of referred symptomatic patients to the hospital. Still, it was consistent with the difference in the proportion of patients with severe lung injury and unlikely to reflect inconsistency between the centres. Our study could definitely benefit from information on the time from onset of the disease to visit the triage centre and underlying medical conditions. Still, unfortunately, this information was not collected.

Another limitation is related to the self-reported vaccination status collected in our study. As a result, possible misclassification could have biased study results. However, most of the vaccinated study participants received their doses in the same year, so we believe the recall bias was insignificant. Furthermore, we consider the population-based proportion of possible fake vaccinations negligible in July 2021, as the most strict control measures were introduced in St. Petersburg later in 2021.

The data we used in our study was collected at the triage centres and not in hospitals. Even though all patients triaged for inpatient treatment were followed-up by the LDCT centre’s personnel from the moment they were referred to the hospital till hospital admission, we do not know the length or the outcome of the hospital admission itself. It is also possible that the patients triaged for outpatient treatment were later admitted. Our study covers only one point in their disease course and traces only the decisions made in the triage centres.

Finally, misclassification could occur when patients with chronic lung diseases and SARS-CoV-2 infection become misdiagnosed with COVID-19 pneumonia. We believe that this misclassification would be non-differential and could underestimate VE. However, future effectiveness studies will benefit from past medical history collected.

Additional observational studies are needed to assess the vaccination effectiveness against SARS-CoV-2 infection and COVID-19 associated death. Given the limitations of our data, we were only able to estimate the protective effect against COVID-19 severe disease. However, it is safe to assume that those outcomes pose as a surrogate for COVID-19-associated death. Our study does not provide any information on the vaccine safety, but at least for Gam-COVID-Vac, the safety data are available from independent sources [10, 11].

## Conclusions

We showed that COVID-19 vaccination is effective against referral to hospital in patients with symptomatic SARS-CoV-2 infection in St. Petersburg, Russia. The protection against hospital admission is probably mediated through protection against lung injury associated with COVID-19. Real-world evidence on the VE against COVID-19 should be integrated into population-based vaccination programmes to negotiate the balance of benefits and harms of this effective COVID-19 control measure, which is likely gaining even more importance in the light of the gradual ceasing of non-pharmaceutical interventions against COVID-19 and calls for a “return to normality”.

## Supplementary Information

**Additional file 1:** **Figure A1. **Vaccine effectiveness against referral to hospital, according to age. **Figure A2. **Probability of any lung injury, according to age and vaccination status. **Figure A3. **Vaccine effectiveness against any lung injury, according to age.

## Data Availability

All analyses were conducted in R; study data and code is available online https://github.com/eusporg/spb_covid_study20).
