# Supplementary material for: Vaccine effectiveness against referral to hospital after SARS-CoV-2 infection in St. Petersburg, Russia, during the Delta variant surge: a test-negative case-control study
Source: BMC Med. 2022 Sep 20;20:312. doi: 10.1186/s12916-022-02509-8 (PMC9484844; doi:10.1186/s12916-022-02509-8)
Supplement: Supplementary file 1 — Additional file 1: Figure A1. Vaccine effectiveness against referral to hospital, according to age. Figure A2. Probability of any lung injury, according to age and vaccination status. Figure A3. Vaccine effectiveness against any lung injury, according to age. [file 12916_2022_2509_MOESM1_ESM.pdf]

Supplementary materials

Vaccine effectiveness against referral to hospital after SARS-CoV-2 infection in St. Petersburg, Russia during the Delta variant surge: a test-negative case-control study

Anton Barchuk, Mikhail Cherkashin, Anna Bulina, Natalia Berezina, Tatyana Rakova, Darya Kuplevatskaya, Oksana Stanevich, Dmitriy Skougarevskiy, Artemiy Okhotin.

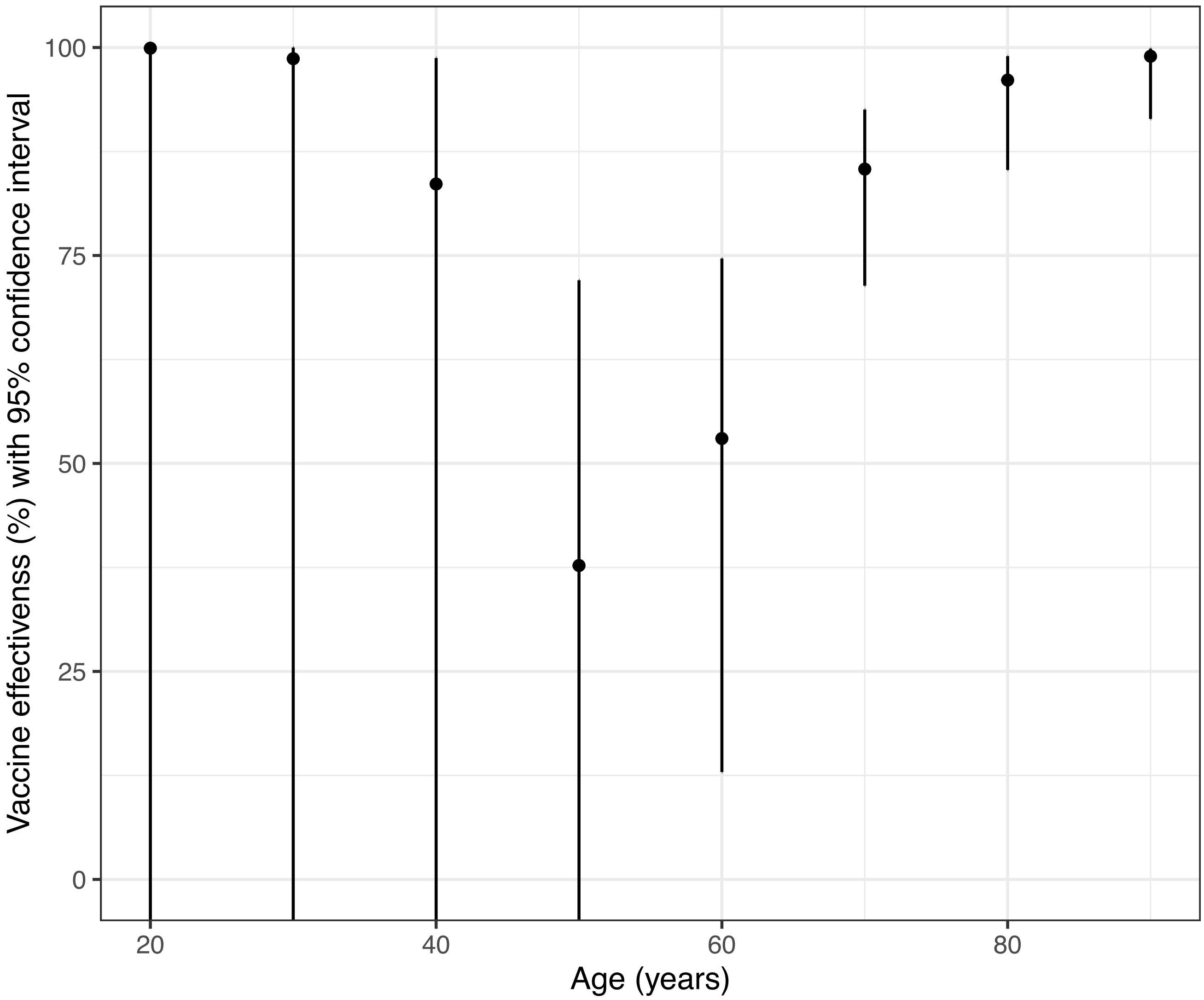

Figure A1. Vaccine effectiveness against referral to hospital, according to age.

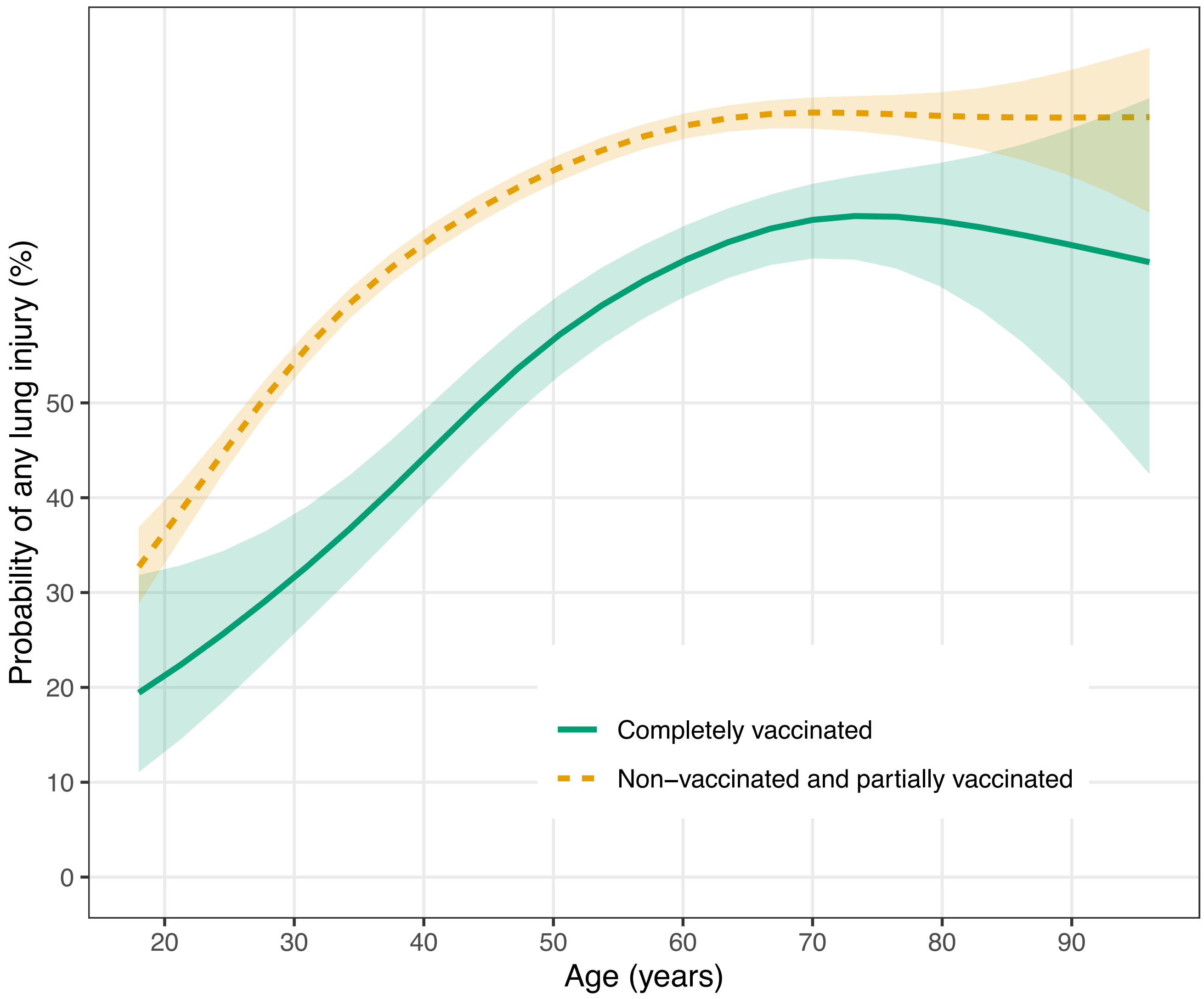

Figure A2. Probability of any lung injury, according to age and vaccination status (shaded areas are 95% CI).

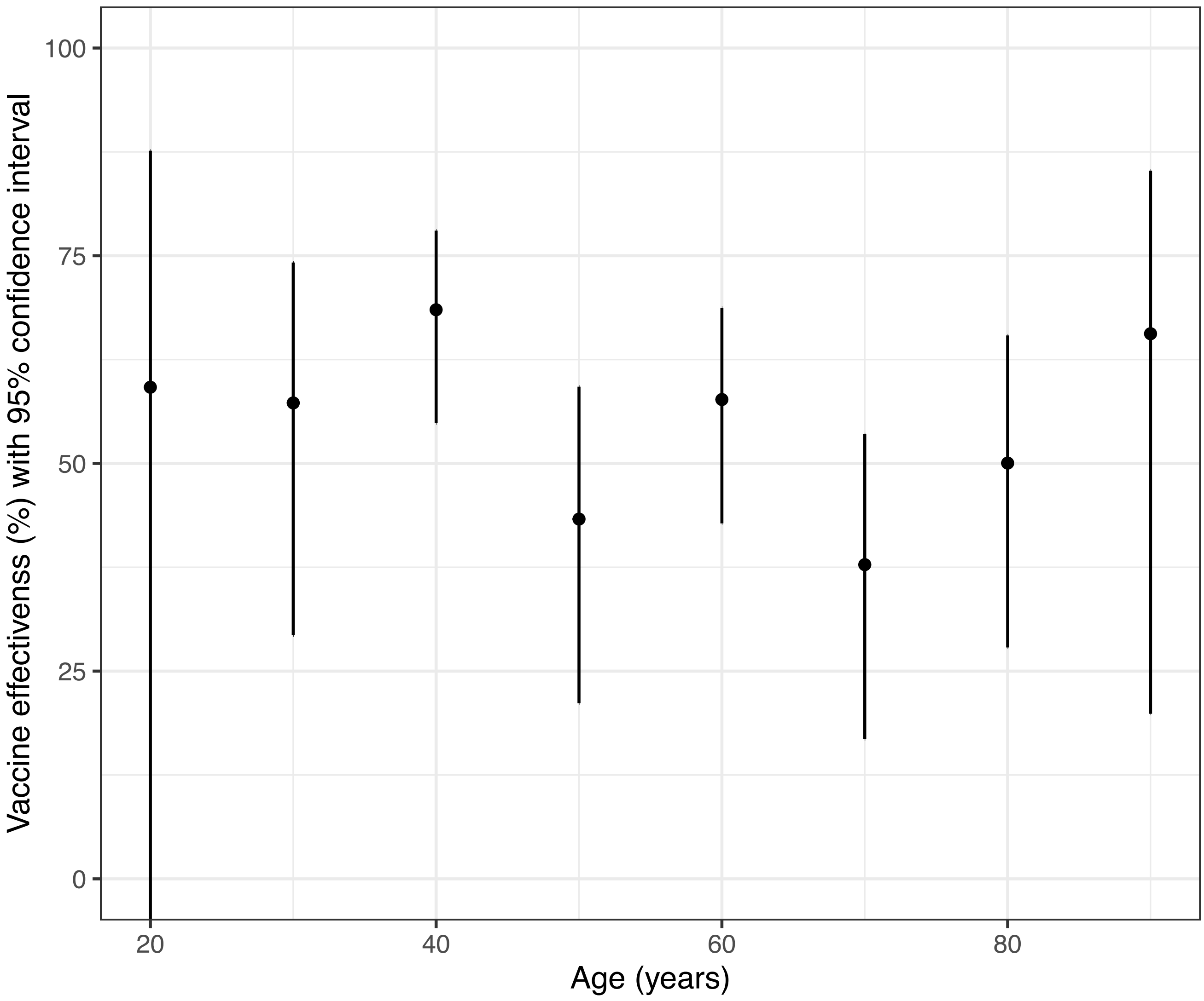

Figure A3. Vaccine effectiveness against any lung injury, according to age.
